# Feasibility and Safety of Physical Exercise to Preserve Bone Health in Men With Prostate Cancer Receiving Androgen Deprivation Therapy: A Systematic Review

**DOI:** 10.1093/ptj/pzab288

**Published:** 2021-12-23

**Authors:** Maribel Cagliari, Barbara Bressi, Maria Chiara Bassi, Stefania Fugazzaro, Giuseppe Prati, Cinzia Iotti, Stefania Costi

**Affiliations:** 1 Department of Surgery, Medicine, Dentistry and Morphological Sciences, University of Modena and Reggio Emilia, Modena, Italy; 2 Department of Biomedical, Metabolic and Neural Sciences, University of Modena and Reggio Emilia, Modena, Italy; 3 Department of Neuromotor Physiopathology and Rehabilitation Medicine, Physical Medicine and Rehabilitation Unit, Azienda USL–IRCCS di Reggio Emilia, Reggio Emilia, Italy; 4 Medical Library, Azienda USL–IRCCS di Reggio Emilia, Reggio Emilia, Italy; 5 Department of Oncology and Advanced Technologies, Oncology Unit, Azienda USL–IRCCS di Reggio Emilia, Reggio Emilia, Italy; 6 Department of Oncology and Advanced Technologies, Radiotherapy Unit, Azienda USL–IRCCS di Reggio Emilia, Reggio Emilia, Italy; 7 Scientific Directorate, Azienda USL–IRCCS di Reggio Emilia, Reggio Emilia, Italy

**Keywords:** Accidental Falls, Bone Density, Bone Health, Exercise, Prostatic Neoplasms

## Abstract

**Objective:**

Men with prostate cancer (PCa) receiving androgen deprivation therapy (ADT) experience the loss of bone mineral density (BMD) and lean body mass, which can increase their risk of falls and fractures. Physical exercise programs with appropriate components and dosage are suggested to preserve BMD and muscle strength, thereby potentially reducing accidental falls and fractures and associated morbidity and mortality. These benefits can be obtained if exercise programs are feasible and safe and if patient adherence is adequate. This systematic review investigates the feasibility and safety of exercise programs aimed at preventing the risk of accidental falls and fractures and BMD loss in men with PCa undergoing ADT.

**Methods:**

MEDLINE, Embase, CINAHL, and the Cochrane Library were searched from database inception to June 7, 2021. Randomized controlled trials were included when they analyzed the feasibility and safety of experimental exercise programs targeting bone health in men with PCa receiving ADT. Two reviewers independently selected the studies, assessed their methodological quality, and extracted the data. Exercise feasibility was measured through recruitment, retention, and adherence rates. Exercise safety was measured through the number, type, and severity of adverse events. Furthermore, the components, setting, intensity, frequency, and duration of exercise programs were extracted.

**Results:**

Ten studies were included, with a total of 633 participants. Exercise consisted of a combination of aerobic, resistance, and impact-loading exercise or football training. Exercise is feasible in men with PCa undergoing ADT, although football training should be prescribed with caution for safety reasons.

**Conclusion:**

Multicomponent exercise programs targeting bone health seem feasible and safe in this population; however, adverse events should be systematically documented according to current guidelines.

**Impact:**

The study shows that men with PCa receiving ADT can safely perform exercise programs to preserve bone health and supports that those programs should become part of lifestyle habits.

**Lay Summary:**

Men with PCa who are receiving ADT can safely perform exercise programs to preserve bone health and should make exercise an important part of their lifestyle.

## Introduction

Prostate cancer (PCa) is the most diagnosed cancer among men worldwide,^1^ and androgen deprivation therapy (ADT) is the first line of treatment in metastatic or advanced stages of this disease.^2^ Nevertheless, ADT causes numerous side effects that can worsen the patient’s quality of life,^3^ such as an increase in cardiovascular disease and metabolic syndrome^4^^,^^5^ and the loss of bone mineral density (BMD) and of muscle strength.^6^^,^^7^ These musculoskeletal alterations contribute to sarcopenia, osteoporosis, and frailty,^8^ which are predictors of accidental falls and fractures in this population,^9–11^ with a significant impact on health-related quality of life, hospitalization, and mortality.^12^

As exercise is well tolerated and safe in cancer survivors,^13^^,^^14^ preliminary evidence supports the introduction of physical exercise programs to improve the clinical and functional outcomes in this population.^15^

More specifically, in men with PCa treated with ADT, exercise has the potential to reduce several of the side effects of ADT, such as the loss of muscle strength, muscle mass, and physical function.^16^ Moreover, exercise programs specifically targeting bone health could preserve BMD.^17^ Altogether, these outcomes may also reduce the risk of accidental falls and fractures, although this effect must still be proven.^18^ However, in order to produce benefits on the musculoskeletal system, exercise should be performed over the long term and at the appropriate dosage.^17^^,^^19^ In this respect, a trend toward becoming less physically active has been documented in older adults,^20^ and several factors may affect patients’ long-term adherence to the prescribed exercise regimen,^21^ such as the side effects of cancer treatments.^22^ In fact, only 41.9% of men with PCa perform the recommended amount of exercise, with greater inactivity for individuals treated with ADT,^23^ whose adherence to experimental exercise has recently been estimated to be as low as 30% to 40%.^24^ However, adherence to exercise may increase when appropriate and acceptable exercise modalities are proposed.^20^ Thus, although adequate exercise programs for men with PCa receiving ADT have the potential to preserve BMD and muscle strength, thereby theoretically reducing the risk of accidental falls and fractures,^18^ this potential cannot be reached if these programs are not sufficiently feasible and safe.

Therefore, this systematic review aimed to investigate the feasibility and safety of physical exercise targeting bone health to prevent BMD loss and accidental falls and fractures in individuals with PCa undergoing ADT. We also aimed to describe the type of exercise (components, setting, intensity, frequency, duration) that can be implemented to preserve bone health in this population.

## Methods

This systematic review was carried out following the Preferred Reporting Items for Systematic Reviews and Meta-Analyses (PRISMA) guidelines.^25^ The study protocol was registered with the International Prospective Register of Systematic Reviews (PROSPERO, number CRD42020163416).

### Data Sources and Searches

A comprehensive search was conducted on MEDLINE, Embase, CINAHL, and the Cochrane Library from their inception until June 7, 2021. The search strategy included terms related to exercise, prostatic neoplasms, androgen antagonists, and associated synonyms (the full search strategy is presented as Suppl. Appendix).

Hand searching of reference lists of the included original studies was undertaken, and the authors of published protocols were contacted to ask for any preliminary results.

### Study Selection

We included randomized controlled trials (RCTs) that met the following eligibility criteria: (1) participants—men with PCa undergoing ADT; (2) intervention—supervised and/or unsupervised exercise programs targeting bone health to prevent BMD loss and accidental falls and fractures; (3) comparison—standard care alone or with placebo; and (4) outcome—feasibility and safety of an experimental exercise program.

Feasibility was estimated based on recruitment and retention rates and on the patients’ adherence to the experimental interventions.^26^ The recruitment rate was calculated as the ratio between randomized participants and individuals assessed for eligibility, and the retention rate was calculated as the ratio between the participants that completed the study and those randomized. Patients’ adherence to the experimental intervention was calculated as the ratio between the number of exercise sessions attended and those planned.

Safety was estimated based on the number and type of adverse events (AEs) reported in the original studies. For the purposes of this systematic review, an AE is any unfavorable symptom or disease that occurred that may or may not be considered related to the intervention experimented (adapted from CTCAE Version 5.0).^27^

We excluded studies where exercise was not the key part of the experimental intervention, that is, any trial focusing chiefly on nutritional, educational, and/or counseling activities.

### Data Extraction and Quality Assessment

Two investigators (B.B., M.C.) screened the title and abstract of the records retrieved and reviewed the full texts using predetermined eligibility criteria. Any disagreement was resolved by discussion and consensus.

Two reviewers (B.B., M.C.) independently assessed the quality of the included studies using the Physiotherapy Evidence Database (PEDro) score,^28^ which is an 11-item checklist to assess the internal validity of an RCT. Each trial is scored out of 10, where a score of 9 or more corresponds to excellent quality, a score from 6 to 8 corresponds to good quality, a score from 4 to 5 corresponds to fair quality, and a score less than 4 corresponds to poor quality.^29^ Disagreements were resolved by consensus with a third reviewer (S.C.). A priori, we decided not to exclude studies from the analyses based on the quality assessment.

**Figure 1:**
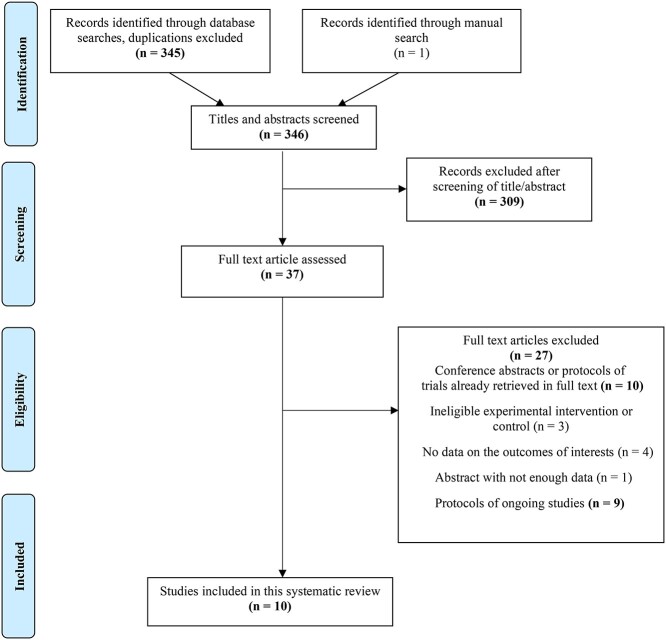
Preferred Reporting Items for Systematic Reviews and Meta-analyses (PRISMA) flow diagram of search and study selection.

### Data Synthesis and Analysis

Two investigators (B.B., M.C.) independently extracted the following data from the included studies: inclusion criteria for participants and sample size, characteristics of the exercise program (setting, type, frequency, intensity, modality), supplementary intervention (nutrition, education, counseling, etc.), comparisons (standard care and placebo, if any), feasibility outcomes (recruitment, retention, and adherence rate**s**), safety outcomes (number, type, and severity of AEs related or unrelated to the intervention), efficacy outcomes (number of falls and fractures and BMD value), and follow-up duration. A detailed description of each exercise component was collected. In the case of missing data, the corresponding authors were contacted (at least 3 attempts) to obtain the desired information.

## Results

### Study Selection

The electronic search strategy identified 345 records, excluding duplicates. Through manual searching, we retrieved 1 more record, for a total of 346. We excluded 309 records based on their title or abstract, assessed the remaining 37 records in full text, and excluded 27 of them for the following reasons: 3 conference abstracts^30–32^ and 7 study protocols^33–39^ were duplicates of full texts retrieved^40–48^; 1 study design experimented an intervention chiefly focusing on education,^49^ 2 others compared different active intervention arms^50^^,^^51^; and 4 studies did not report data regarding the outcomes of interest, that is, they did not report any measure of feasibility or safety of exercise.^46^^,^^47^^,^^52^^,^^53^ Further, 10 studies were excluded because they reported insufficient data for analysis^54^ or were protocols of ongoing, unpublished studies.^55–63^ We contacted the authors to collect any preliminary results (minimum 3 attempts), but they had no data to share yet.

Therefore, 10 studies met the inclusion criteria,^40–45^^,^^48^^,^^64–66^ providing data from 9 RCT designs, 1 of which yielded 2 published studies^44^^,^^45^ reporting data collected on the same sample at 2 different follow-ups (Fig. 1).

### Quality Assessment

The quality of the RCTs included is reported in Table 1. Blinding of participants and therapists was not possible due to the type of intervention. All the included studies reported random allocation, similar groups at baseline, differences between groups, and point estimate variability. Most of the included studies reported the intention-to-treat analysis,^40–43^^,^^48^^,^^64^ and 5 of the included studies reported concealed allocation,^40^^,^^43–45^^,^^65^ adequate follow-up (>85%),^40^^,^^44^^,^^48^^**,**^^64^^,^^66^ and blinding of assessors.^42^^,^^43^^,^^48^^,^^64^^,^^65^ Overall, 7 studies were deemed as good quality^40^^,^^42–44^^,^^48^^,^^64^^,^^65^ and 3 as fair.^40^^,^^45^^,^^66^ The 2 published studies by Uth et al yielded different PEDro scores due to the lower dropout rate at the 3-month follow-up^44^ compared with the dropout rate at the 8-month follow-up.^45^

**Table 1:** Physiotherapy Evidence Database (PEDro) Score of the Included Studies^a^

**Study**	**Random Allocation**	**Concealed Allocation**	**Groups Similar at Baseline**	**Participant Blinding**	**Therapist Blinding**	**Assessor Blinding**	**<15% Dropouts**	**Intention- to-Treat Analysis**	**Between Difference Reported**	**Point Estimate and Variability Reported**	**Total (0 to 10)**
Cormie et al^40^ (2015)	Y	Y	Y	N	N	N	Y	Y	Y	Y	7
Dalla Via et al^48^ (2021)	Y	N	Y	N	N	Y	Y	Y	Y	Y	7
Kim et al^65^ (2018)	Y	Y	Y	N	N	Y	N	N	Y	Y	6
Lam et al^66^ (2020)	Y	N	Y	N	N	N	Y	N	Y	Y	5
Newton et al^41^ (2019)	Y	N	Y	N	N	N	N	Y	Y	Y	5
Nilsen et al^43^ (2015)	Y	Y	Y	N	N	Y	N	Y	Y	Y	7
Taaffe et al^42^ (2019)	Y	N	Y	N	N	Y	N	Y	Y	Y	6
Uth et al^44^ (2016)	Y	Y	Y	N	N	N	Y	N	Y	Y	6
Uth et al^45^ (2016)	Y	Y	Y	N	N	N	N	N	Y	Y	5
Winters-Stone et al^64^ (2014)	Y	N	Y	N	N	Y	Y	Y	Y	Y	7

^*^a^*^Y = yes, N = no.

### Characteristics of the Studies

The characteristics of the RCTs included in this review are shown in Table 2. The studies, published between 2014 and 2021**,** were conducted in Europe,^43–45^ Australia,^40–42^^,^^48^^,^^66^ the United States,^64^ and Asia^65^ and promoted by university hospitals^40^^,^^42^^,^^43^^,^^65^^,^^66^ or specialized prostate cancer centers.^41^^,^^44^^,^^45^^,^^48^ Two were crossover designs^41^^,^^42^; for the purposes of this review, we considered the data of the first follow-up, before the switch of the treatments, which in both cases was fixed at 6 months. Because 1 RCT compared 2 active interventions with 1 control (impact loading plus resistance exercise vs control, and aerobic plus resistance exercise vs control), we considered both the comparisons for the purposes of this review.^41^ The follow-up period varied from 6 weeks^66^ to 12 months^48^^,^^64^^,^^66^ after the baseline assessment. All the included studies investigated the effectiveness of exercise to prevent BMD loss; none registered accidental falls and fractures.

**Table 2:** Study Characteristics^a^

**Study**	**Country**	**Participants**	**Main Exclusion Criteria**	**Intervention**	**Outcome Measures**
Cormie et al^40^ (2015)	Australia	• Local. and metastatic PCa treated with ADT • N° tot = 63 IG = 32; CG = 31 • Mean age, y = 68.3*^b^* (range: 46–80) Time on ADT, mean (SD): IG = 6.2 (1.6) d; CG = 5.6 (2.0) d	• Bone metastasis • Previous treatment with ADT	IG = supervised exercise program involving aerobic and resistance exercise sessions CG = standard care	• BMD = areal bone mineral density of whole body, lumbar spine (L2–L4), femoral neck • Follow-up = 3 mo
Dalla Via et al^48^ (2021)	Australia	• Local and metastatic PCa treated with ADT • N° tot = 70 IG = 34; CG = 36 • Mean age, y = 71.0 (range: 50–85) • Time on ADT, mean (IQR): IG = 8.0 mo (4.0–22.0); CG = 13.0 mo (8.0–24.0)	None	IG = supervised and unsupervised resistance exercise plus weight-bearing impact exercise combined with multinutrient supplementation CG = standard care	• BMD = areal bone mineral density of total hip, lumbar spine (L1–L4), femoral neck • Feasibility = retention and adherence • Safety = adverse events related to exercise • Follow-up = 6 mo, 12 mo
Kim et al^65^ (2018)	South Korea	• Local and metastatic PCa treated with ADT • N° tot = 51: IG = 26; CG = 25 • Mean age, y = 70.8 (range: 20–80) • Time on ADT, mean (SD): IG = 22.5 (26.5) mo; CG = 21.6 (19.1) mo	• Bone metastasis • Osteoporosis	IG = unsupervised weight-bearing and resistance exercise with optional program (stabilization/balance exercise + circuit resistive calisthenics) CG = stretching exercise	• BMD = total hip, lumbar spine (L1–L4), femoral neck • Feasibility = retention and adherence • Safety = adverse events related to exercise • Follow-up = 6 mo
Lam et al^66^ (2020)	Australia	• Local and metastatic PCa treated with ADT • N° = 25: IG = 13; CG = 12 Mean age, y = 70.5*^b^* • Time on ADT: IG = 0 d; CG = 0 d	• Previous treatment with ADT (within the last 12 mo)	IG = home-based progressive resistance training program CG = standard care	• BMD = femoral neck and lumbar spine • Feasibility = retention and adherence • Safety = adverse events related to exercise • Follow-up = 6 wk, 6 mo, 12 mo
Newton et al^41^ (2019)	Australia	• Local and metastatic PCa treated with ADT • N° tot = 154 IG = 57 (ImpRe); = 50 (AerRe); CG = 47 • Mean age, y (SD; range) = 69.0 (9.0; 43–90) • Time on ADT, mean (IQR): IG (ImpRe) = 3.0 mo (2.0–4.0); IG (AerRe) = 3.0 mo (2.0–4.0); CG = 2.0 mo (2.0–3.5)	• Bone metastasis	IG (ImpRe) = supervised and unsupervised impact-loading and resistance exercise IG (AerRe) = supervised aerobic and resistance exercise CG = standard care	• BMD = whole body, total hip, lumbar spine (L2–L4), femoral neck, trochanter • Follow-up = 6 mo
Nilsen et al^43^ (2015)	Norway	• Local and metastatic PCa treated with ADT • N° tot = 58 IG = 28; CG = 30 • Mean age, y = 66.0 (range: 54–76) • Time on ADT, mean (SD): IG = 9.0 (1.6) mo; CG = 9.0 (1.8) mo	• Osteoporosis	IG = supervised and unsupervised high-load strength program CG = standard care	• BMD = areal bone mineral density of whole body, total hip, total lumbar spine, femoral neck, trochanter • Feasibility = adherence • Follow-up = 4 mo
Taaffe et al^42^ (2019)	Australia	• Local PCa treated with ADT • N° tot = 104 IG = 54; CG = 50 • Mean age, y = 68.2*^b^* (range: 48–84) • Time on ADT, mean (SD): IG = 6.4 (2.1) d; CG = 5.7 (1.9) d	• Osteoporosis • Previous treatment with ADT	IG = supervised resistance + aerobic + impact exercise sessions CG = standard care	• BMD = whole body, total hip, lumbar spine • Follow-up = 6 mo
Uth et al^44^ (2016)	Denmark	• Local and metastatic PCa treated with ADT • N° tot = 57 IG = 29; CG = 28 • Mean age, y = 67.0 Time on ADT, mean (IQR): IG = 12.5 mo (9.5–27.8); CG = 18.7 mo (9.4–35.0)	• Osteoporosis	IG = football training CG = standard care	• BMD = areal bone mineral density of whole body, total hip, total lumbar spine, femoral neck, trochanter • Feasibility = adherence • Safety = adverse events • Follow-up = 3 mo
Uth et al^45^ (2016)	Denmark	(Same sample as in the study by Uth et al^44^)	(Same sample as in the study by Uth et al^44^)	(Same sample as in the study by Uth et al^44^)	• BMD = areal bone mineral density of whole body, total hip, total lumbar spine, femoral neck, trochanter • Feasibility = adherence • Safety = adverse events • Follow-up = 8 mo
Winters-Stone et al^64^ (2014)	USA	• Local and metastatic PCa treated with ADT • N° tot = 51 IG = 29; CG = 22 • Mean age, y = 70.2 • Time on ADT, mean (SD): IG = 39.0 (36.1) mo; CG = 28.5 (29.2) mo	• Bone metastasis • Osteoporosis	IG = supervised impact and resistance training CG = stretching exercise	• BMD = total hip, lumbar spine (L1–L4), femoral neck, greater trochanter • Follow-up = 6 mo, 12 mo

^*^a^*^ADT = androgen deprivation therapy; AerRe = aerobic + resistance training; BMD = bone mineral density; CG = control group; IG = intervention group; ImpRe = impact + resistance training; IQR = interquartile range; N tot = total number of participants; PCa = prostate cancer.

^*^b^*^Estimated mean age of participants.

### Participants

The RCTs included 633 men with local or metastatic PCa undergoing ADT whose average age ranged from 66.0 to 71.0 years (Tab. 2). The sample size ranged from 25 to 154 participants. Overall, 352 men were allocated to experimental exercise programs and 281 to the control group.

Five studies^40^^,^^44^^,^^45^^,^^48^^,^^64^^,^^65^ reported the average time from diagnosis of PCa to enrollment, ranging from 15 to 79 months in participants allocated to experimental exercise, and from 10 to 76 months in participants allocated to the control group. Participants had been previously treated for cancer by prostatectomy,^40^^,^^42^^,^^44^^,^^45^^,^^48^^,^^65^ radiation therapy,^40^^,^^43–45^^,^^48^^,^^64–66^ and/or chemotherapy.^40^^,^^48^^,^^64^ Concomitant cancer treatments were generally allowed, and in some cases, ADT associated with radiation therapy was documented during the participation in the trial.^40–42^^,^^48^ Only 5 studies^43–45^^,^^48^^,^^65^^,^^66^ reported data on cancer stage, which ranged from stage I to IV according to the TNM classification.

The most frequent exclusion criteria to participation were: (1) bone metastasis,^40^^,^^41^^,^^64^^,^^65^ (2) osteoporosis,^42–45^^,^^64^^,^^65^ and (3) previous treatment with ADT.^40^^,^^42^^,^^66^

### Feasibility Outcomes: Recruitment, Retention, and Adherence Rates

The data for recruitment, retention, and adherence rates are reported in Table 3.

**Table 3:** Feasibility Outcomes: Recruitment, Retention, and Adherence Rates^a^

**Study**	**Recruitment**	**Retention**	**Dropouts**	**Adherence**
Cormie et al^40^ (2015)	• June 2011 to October 2012 • Recruited: 50.0% • Recruitment strategy: clinician referral	• Study: 87.3%*^b^* IG: 96.9%*^b^* CG: 77.4%*^b^*	• Study: n = 8 IG: n = 1 CG: n = 7	• IG: 96.3%
Dalla Via et al^48^ (2021)	• April 2014 to November 2017 • Recruited: 32.7% • Recruitment strategy: clinician referral, advertisements, and support group	• Study (6 mo): 91.4%*^b^* IG: 97.1%*^b^* CG: 86.1%*^b^*	• Study: n = 6 IG: n = 1 CG: n = 5	• IG: 65% (SE) 49% (UE)
		• Study (12 mo): 86.0% IG: 91.2%*^b^* CG: 80.6%*^b^*	• Study: n = 4 IG: n = 2 CG: n = 2	
Kim et al^65^ (2018)	• May 2013 to September 2015 • Recruited: 14.0% • Recruitment strategy: screening of outpatients of urology units	• Study: 80.4%*^b^* IG: 88.5%*^b^* CG: 72.0%*^b^*	• Study: n = 10 IG: n = 3 CG: n = 7	• IG: 64.8% (RE); 84.7% (WBE) CG: 40%
Lam et al^66^ (2020)	• >2 y • Recruited: 62.5% • Recruitment strategy: clinician referral	• Study (6 wk): 100.0%*^b^* IG: 100.0%*^b^* CG: 100.0%*^b^*	• Study: n = 0 IG: n = 0 CG: n = 0	• IG: 100%
		• Study (6 mo): 92.0%*^b^* IG: 92.3%*^b^* CG: 100.0%*^b^*	• Study: n = 1 IG: n = 1 CG: n = 0	• IG: 82.5%
		• Study (12 mo): 80.0%*^b^* IG: 76.9%*^b^* CG: 83.3%*^b^*	• Study: n = 4 IG: n = 2 CG: n = 2	• IG: 77.9%
Newton et al^41^ (2019)	• 2009–2012 • Recruited: 58.1% • Recruitment strategy: clinician referral	• Study: 76.6%*^b^* IG: 73.7*^b^* (ImpRes) 86.0%*^b^* (AerRes) CG: 70.2%^b^	• Study: n = 36 IG: n = 15 (ImpRes) n = 7 (AerRes) CG: n = 14	• IG: 65% (ImpRes), 70% (AerRes)
Nilsen et al^43^ (2015)	• December 2008 to December 2011 • Recruited: 14.0% • Recruitment strategy: screening of oncology and urology units	• Study: 84.5%*^b^* IG: 78.6%*^b^* CG: 90.0%*^b^*	• Study: n = 9 IG: n = 6 CG: n = 3	• IG: 88% (LB) 84% (UB)
Taaffe et al^42^ (2019)	• August 2013 to April 2015 • Recruited: 47.5% • Recruitment strategy: clinician referral	• Study: 81.7%*^b^* IG: 88.9%*^b^* CG: 74.0%*^b^*	• Study: n = 19 IG: n = 6 CG: n = 13	• IG: 79%
Uth et al^44^ (2016)	• February 2012 to September 2013 • Recruited: 73.1% • Recruitment strategy: screening of outpatients of urology units	• Study (3 mo): 86.0%*^b^* IG: 89.7%*^b^* CG: 82.1%*^b^*	• Study: n = 8 IG: n = 3 CG: n = 5	• IG: 76.5%
Uth et al^45^ (2016)		• Study (8 mo): 71.9%*^b^* IG: 72.4%*^b^* CG: 71.4%*^b^*	• Study: n = 8 IG: n = 5 CG: n = 3	• IG: 46.2%
Winters-Stone et al^64^ (2014)	• >2 y • Recruited: 10.9% • Recruitment strategy: clinician referral, enrollment from cancer registries, advertisements, support group, and community events	• Study: 84.0% IG: 90.0% CG: 77.0%	• Study: n = 8 IG: n = 3 CG: n = 5	• IG: 84% (SE); 43% (HE) • CG: 74% (SE); 51% (HE)

^*^a^*^AerRes = aerobic + resistance exercise; CG = control group; HE = home exercise; IG = intervention group; ImpRes = impact + resistance exercise; LB = lower body; RE = resistance exercise; SE = supervised exercise; UB = upper body; UE = unsupervised exercise; WBE = weight-bearing exercise.

^*^b^*^Calculated from the CONSORT diagram of the study.

The recruitment rate for the RCTs included in this review ranged from 10.9%^64^ to 73.1%.^44^^,^^45^ The recruitment period ranged from 12 months^44^^,^^45^ to 43 months.^48^

Recruitment encompassed various modalities, including clinician referral,^40–42^^,^^48^^,^^66^ the screening of inpatients and outpatients of oncology and urology units,^43–45^^,^^65^ or combined strategies that also included enrollment from cancer registries, advertisements, and group/community events.^48^^,^^64^ Most studies enrolled fewer patients than the number planned; only 2 studies were able to recruit the expected sample size.^40^^,^^66^

Overall, the retention rate varied from 71.9%^45^ to 100%.^66^ Most studies (n = 8) reported a retention rate exceeding 80%, which had also been recorded at the 12-month follow-ups.^48^^,^^64^^,^^66^ All but 2 studies^43^^,^^66^ showed a higher retention rate in the intervention group (IG) than in the control group (CG). Overall, 55 men withdrew from the exercise intervention, representing 15.6% of the 352 participants enrolled to the IG. Only 6 men dropped out due to reasons likely related to the intervention: 4 reported exercise-associated pain or muscle strain,^43^^,^^44^ 1 disliked the type of exercise proposed (football),^44^ and another disliked the setting of exercise (clinic).^42^ Moreover, 7 individuals dropped out due to low motivation to exercise.^41^^,^^42^ However, most of the dropouts were among the participants allocated to CG (n = 66; 23.5%). All reasons for dropping out are reported in Table 4.

Adherence rates ranged from 43%^64^ to 96.3%^40^ in the IG and from 40%^65^ to 74%^64^ in the CG. When exercise interventions were supervised,^40–45^^,^^48^^,^^64^ the highest adherence rate was registered for the 3-month aerobic and resistance exercise program (96.3%),^40^ whereas the lowest was registered for the 8-month football training program (46.2%).^45^ Among the RCTs that experimented unsupervised exercise,^41^^,^^43^^,^^48^^,^^64–66^ high adherence was shown when exercise consisted of weight-bearing activities such as walking (84%),^65^ and lower adherence was related to resistance plus impact exercises (49% and 43%).^48^^,^^64^ Two studies did not report data of adherence to unsupervised exercise.^41^^,^^43^ Two study designs implemented a stretching intervention for men allocated to CG. The adherence rate to this active control was equal to 74% when supervised, and between 40% and 51% when unsupervised.^64^^,^^65^ Printed exercise booklets^41^ and 10-minute telephone sessions^65^ were strategies used by some studies to facilitate adherence in the CG.

### Safety Outcome

The safety of interventions is summarized in Table 5. Although all the studies included in this review monitored the AEs associated with experimental exercise, only 3 studies described how AEs were recorded,^44^^,^^45^^,^^48^^,^^65^ and 2 reported how their severity was defined.^44^^,^^45^^,^^65^ Uth et al^44^^,^^45^ complied with existing guidelines,^67^ and Kim et al^65^ recorded falls, injuries, and exercise-associated symptoms as AEs attributable to exercise. Overall, 30 AEs were related to exercise,^43–45^^,^^48^^,^^66^ 3 were classified as severe (2 fibula fractures and 1 partial Achilles tendon rupture),^44^ and 27 were minor musculoskeletal AEs.^43^^,^^44^^,^^48^^,^^66^ In the other studies, no AEs were reported.

**Table 4:** Reasons for Dropping Out

**Reason**	**Intervention Group (n)**	**Control Group (n)**
Became ineligible	4	6
Health issues	27	19
Lost to follow-up	1	6
No longer interested in participating	7	10
Personal issues	5	7
Time constraints	3	4
Too far to travel	—	2
Wanted to exercise at home	1	—
Wanted to start exercising	—	8
Death	3	2
Other	4	2

**Table 5:** Details of Exercise Programs and Safety Outcomes^a^

**Study**	**Detailed Intervention IG**	**Detailed Intervention CG**	**Adverse Events**
Cormie et al^40^ (2015)	• Intervention period: 3 mo • Supervised exercise in exercise clinic: - Aerobic exercise: 70%–85% max HR × 20–30 min - Resistance exercise: 6–12 RM × 1–4 sets Modality: Group Each session: 60 min (with warm-up and cool-down), 2 d/wk Supplemental exercise: home-based aerobic activity to accumulate 150 min/wk	No intervention	• Referred to exercise: IG: 0; CG: 0 • Not referred: IG: 1; CG: 0
Dalla Via et al^48^ (2021)	• Intervention period: 12 mo • Supervised exercise in health and fitness facility (gym): - Aerobic exercise: 55%–75% max HR × 15–25 min - Resistance exercise: 3–8 RPE, 2 sets × 8–15 reps - eight-bearing, impact exercise: 1–9 times BW, 3 sets × 10–20 reps - Balance/functional exercise: 2 sets of 30–60 s or for given number of reps - Core stability exercise: 2 sets × 10–15 reps Modality: NR Each session: 60 min (with warm-up and cool-down), 2 d/wk (after 6 mo only 1 session was supervised) • Unsupervised exercise in home setting: Similar to supervised one but used BW and resistance bands Modality: Individual Each session: 20–60 min, 1 d/wk	No intervention	• Referred to exercise: IG: 21; CG: 0*^b^* • Not referred: IG: 3; CG:5
Kim et al^65^ (2018)	• Intervention period: 6 mo • Unsupervised exercise in home setting: - Resistance exercise: 0%–10% BW × 2–3 sets × 8–15 reps - Weight-bearing exercise: 11–15 RPE × 20–30 min Modality: Individual Each session: started with a warm-up, 2–5 d/wk of resistance exercise; 3–5 d/wk of weight-bearing exercise • Optional program: stabilization/balance exercise + circuit resistive calisthenics × 2–5 d/wk	• Intervention period: 6 mo • Unsupervised stretching in home setting: - Whole-body stretching (lying, sitting, standing) Modality: individual • Each session: 20 min, 3–5 d/wk	• Referred to exercise: IG: 0; CG: 0 • Not referred: • IG: 1; CG:0
Lam et al^66^ (2020)	• Intervention period: 12 mo • Unsupervised exercise in home setting: - Resistance exercise: 8–12 RM × 3 sets Modality: Individual Each session: 40 min, 3 d/wk	• No intervention	• Referred to exercise: IG: 1; CG: 0 • Not referred: IG:• 0; CG:1
Newton et al^41^ (2019)	ImpRes • Intervention period: 6 mo • Supervised exercise in exercise clinic - Resistance exercise: 6–12 RM × 2–4 sets - Impact exercise: 3–5 times BW × 2–4 sets Modality: Group Each session: 60 min (with warm-up and cool-down) 2 d/wk • Unsupervised exercise in home setting - Impact exercise: 2–4 sets Modality: Individual Each session: 2 d/wk AerRes • Intervention period: 6 mo • Supervised exercise in exercise clinic: - Resistance exercise: 6–12 RM × 2–4 sets - Aerobic exercise: 60%–85% max HR × 20–30 min Modality: Group Each session: 60 min (with warm-up and cool-down), 2 d/wk • upplemental exercise: home-based aerobic activity to accumulate 150 min/wk	• Printed booklet with information about exercise	• Referred to exercise: IG: 0 (ImpRes); IG: 0 (AerRes); CG: 0 • Not referred: IG: 8 (ImpRes); IG: 2 (AerRes); CG: 4
Nilsen et al^43^ (2015)	• Intervention period: 4 mo • Supervised exercise in clinic exercise: - Resistance exercise: 6–10 RM × 1–3 sets Modality: Group Each session: 2 d/wk • Unsupervised exercise in clinic exercise: - Resistance exercise: 80%–90% of 10 RM × 2–3 sets ×10 rep Modality: Group or Individual Each session: midweek session (1 d/wk)	• Encouraged to maintain their habitual physical activity level	• Referred to exercise: IG: 3; CG: 0 • Not referred: IG: 3; CG: 3
Taaffe et al^42^ (2019)	• Intervention period: 6 mo • Supervised exercise in exercise clinic: - Aerobic exercise: 60%–85% max HR × 25–40 min - Resistance exercise: 6–12 RM × 2–4 sets - Impact exercise: 3.4–5.2 times BW × 2–4 sets Modality: Group Each session: 60 min (with warm-up and cool-down), 3 d/wk (aerobic and resistance exercise were performed in alternated session days) • Supplemental exercise: home-based aerobic activity + modified impact-loading exercise × 2 d/wk	• No intervention	• Referred to exercise: IG: 0; CG: 0 • Not referred: IG: 3; CG: 7
Uth et al^44^ (2016)	• Intervention period: 3 mo • Supervised exercise on pitch (out/indoors) - Football exercise: 2–3 sets × 15 min Modality: Group Each session: 45–60 min (with warm-up), 2–3 d/wk	• Encouraged to maintain their habitual physical activity level	• Referred to exercise: IG: 5; CG: 0*^b^* • Not referred: IG: 4; CG: 0
Uth et al^45^ (2016)	• Intervention period: 8 mo • Supervised exercise on pitch (out/indoors) - Football exercise: 3 sets × 15 min Modality: Group Each session: 60 min (with warm-up), 2 d/wk		
Winters-Stone et al^64^ (2014)	• Intervention period: 12 mo • Supervised exercise in exercise clinic: - Resistance exercise: Upper body: 8–15 RM × 1–2 sets × 8–14 reps Lower body: 0%–15% BW × 1–2 sets × 8–12 reps -Impact exercise: 0%–10% BW × 1–10 sets × 10 reps Modality: Group Each session: 60 min, 2 d/wk • Unsupervised exercise in home setting: - Similar to supervised one with resistance bands that replaced weighted vest used in impact exercise Modality: Individual Each session: 60 min, 1 d/wk	• Intervention period: 12 mo • Supervised exercise in exercise clinic - Whole-body stretching and relaxation exercise in a seated or lying position Modality: Group Each session: 60 min, 2 d/wk • Unsupervised exercise in home setting - Similar to supervised one Modality: Individual Each session: 60 min, 1 d/wk	• Referred to exercise: IG: 0; CG: 0 • Not referred: IG: 1; CG: 3

^*^a^*^AerRes = aerobic + resistance exercise; BW = body weight; CG = control group; HR = heart rate; IG = intervention group; ImpRes = impact + resistance exercise; NR = not reported; rep = repetition; RM = repetition maximum; RPE = rate of perceived exertion.

^*^b^*^Adverse events were not monitored in the CG.

In 1 study, the exercise intervention was adapted to meet the needs of 2 men who had knee and shoulder discomfort due to the high workload.^64^ However, a large number of AEs not attributable to exercise were reported as generic health issues/hospitalization (n = 13 IG, n = 17 CG),^41–43^^,^^45^^,^^48^^,^^64^^,^^65^^,^^66^ injury/accident (n = 8 IG, n = 2 CG),^41–43^ and death (n = 3 IG, n = 2 CG).^41^^,^^48^^,^^64^ In a few cases, AEs were reported as pain (n = 1 CG),^43^ fatigue (n = 1 CG),^42^ ADT side effects (n = 1 IG),^40^ and peripheral neuropathy (n = 1 IG).^44^^,^^45^

### Characteristics of Experimental Exercise: Components, Posology, and Setting

Table 5 reports the main features of the exercise program. The duration of exercise varied from 3 months^44^ to 12 months.^48^^,^^64^^,^^66^ Most studies implemented a multicomponent experimental exercise consisting of aerobic exercise associated with resistance exercise^40^^,^^41^^,^^65^ or with impact-loading exercise,^42^ or consisting of resistance exercise and impact-loading exercise.^41^^,^^48^^,^^64^ Two studies implemented a single-component resistance training program,^43^^,^^66^ and another included balance and core stability exercises.^48^

Resistance training consisted of exercises targeting the major upper and lower body muscle groups and involving free weight, weight machines, or resistance bands.^40–43^^,^^48^^,^^64^^,^^65^^,^^66^ Six studies reported that training intensity was progressively increased by 2% to 5%^43^^,^^64^^,^^65^ or 5% to 10% increments,^40–42^ with reference to the individual target defined through a repetition maximum test.^41–43^^,^^64^

Impact exercise consisted of drop jumping activities either alone^64^ or combined with a series of bounding, hopping, skipping, and/or leaping.^41^^,^^42^^,^^48^ The intensity of these activities was set as the percentage of body weight and was progressively increased over time.^41^^,^^42^^,^^48^^,^^64^

Aerobic exercise consisted of weight-bearing activities such as walking or jogging,^40–42^^,^^65^ cycling or rowing on a stationary ergometry,^40–42^^,^^48^ or exercising on a cross-trainer machine.^40^^,^^41^ Aerobic activities were performed for from 15 to 40 minutes, 1 to 2 times per week at the intensity of 55% to 85% of the maximum heart rate^40–42^^,^^48^ or with the aim of reaching 150 minutes per week of moderate-intensity exercise.^65^ Exercise intensity during sessions was frequently monitored by way of a perceived exertion scale, asking individuals to exercise at a level between “somewhat hard” to “hard.”^40^^,^^42^^,^^65^

It should be noted that 1 study implemented football training, which can be considered a combination of aerobic, resistance, and impact exercise.^44^^,^^45^ The intensity of football training was progressively increased both in the number and in the duration of sessions for the first 3 months,^44^ then a maintenance program was undertaken for the following 5 months.^45^

In most cases, the exercise session lasted 40 to 60 minutes and was performed 2 to 3 times a week,^40^^,^^42–45^^,^^48^^,^^64^^,^^66^ and even 4 to 5 times per week.^41^^,^^65^

Frequently, exercise sessions began with warm-up and ended with cool-down exercises or relaxation activities.^40–42^^,^^44^^,^^45^^,^^48^^,^^65^

The exercise sessions were either completely supervised,^40–42^^,^^44^^,^^45^ unsupervised,^65^^,^^66^ or a mix of supervised and unsupervised.^41^^,^^43^^,^^48^^,^^64^ Supervised sessions were administered to groups^40–45^^,^^48^^,^^64^ and performed in exercise clinics^40–43^^,^^64^ or a gym^48^ or in sport settings (natural grass pitch or indoors for football training).^44^^,^^45^ Unsupervised sessions could be implemented individually^41^^,^^45^^,^^64^^,^^66^ or in groups^43^ and were performed at home,^41^^,^^48^^,^^64–66^ at a gym,^48^ or in exercise clinics.^43^

In most studies, the men allocated to the CG were encouraged to engage in exercise or to maintain their habitual physical activity level, whereas 2 studies implemented a full body stretching program for individuals allocated to CG.^64^^,^^65^

### Supplementary Interventions

Several study designs also implemented a supplementary home-based program for men allocated to IG, with a frequency of 2 to 5 times per week: 2 studies proposed aerobic exercise to accumulate 150 minutes per week^40^^,^^41^; 1 proposed a combination of aerobic with impact exercise^42^; 1 study proposed a stabilization/balance exercise and circuit resistive calisthenics.^65^

Some studies also provided the men allocated to experimental exercise programs with educational counseling or educational material regarding exercise,^65^^,^^66^ exercise logs where the men recorded the exercise activities performed individually,^41^^,^^42^^,^^65^^,^^66^ or monthly reminder phone calls.^66^ Moreover, 1 study experimented exercise associated with daily multinutrient supplementation compared with vitamin D only for the control group,^48^ and another study provided calcium and vitamin D supplementation for both the intervention and the control groups.^42^

## Discussion

This systematic review suggests that exercise is feasible and safe in men with PCa undergoing ADT.

Recruitment and adherence rates varied between studies, but the latter was frequently higher in the experimental intervention group than in the control group. Most studies reported a retention rate exceeding 80%,^40^^,^^42–44^^,^^48^^,^^64–66^ with higher rates registered for the intervention groups.^40^^,^^41^^,^^44^^,^^45^^,^^48^^,^^64^^,^^65^ Finally, 30 AEs were associated with experimental exercise^45^^,^^48^^,^^66^ 3 were classified as severe, and all were associated with football training.^44^^,^^45^

It is well known that participation in trials of cancer survivors is a challenge, especially for populations over the age of 65.^68^^,^^69^ The average age of the study samples included in this review was 66 to 71.0 years; 5 of the included studies reached a recruitment rate of close to 50% or over (47.5%–73.1%).^40–42^^,^^44^^,^^45^^,^^66^ Moreover, 7 studies reached 80% of the sample size set a priori.^40–43^^,^^64–66^ The retention rate was quite high in all the studies included. The adherence rate for individuals allocated to IG ranged from 43%^64^ to 96.3%,^40^ suggesting that exercise is feasible in this population.

The feasibility of the experimental exercise may have been influenced by several factors, for example, the recruitment strategy applied. The most successful recruitment strategy seemed to be clinician referral,^40–42^^,^^66^ whereas advertisements and community events did not seem to add any substantial advantage.^48^^,^^64^

The retention rate exceeded 70% in all the included studies; a few individuals (6 of 51) dropped out due to reasons attributable to the experimental exercise, although dropouts were more frequent in the CG. This might suggest that exercise is well tolerated and appreciated in this population, even if men with PCa undergoing ADT are often older, fragile individuals with health issues that might influence participation in exercise. A frequently reported reason for dropping out was the loss of interest in the study: this finding supports the importance of adequate strategies to sustain participants’ interest during the trial (eg, follow-up phone calls, adequate progression of intensity of exercise),^70^ including the proposal of an active control (such as stretching or alternative exercise) to avoid patients dropping out due to their desire to start exercising.

Furthermore, this review shows a higher adherence rate to supervised (range: 46%–96%) rather than to unsupervised exercise (range: 40%–84%), confirming the value of having a supervisor during the training sessions in this population.^71^^,^^72^ However, 2 studies,^65^^,^^66^ which proposed completely unsupervised exercise supported by education material and monitoring of exercise by phone, reported an adherence rate exceeding 60%.

Concerning the safety of exercise, 30 AEs were associated with football training, resistance exercise, and resistance plus impact exercise^43–45^^,^^48^^,^^66^; of these AEs, 3 were severe, all occurring during football training.^44^^,^^45^ Nevertheless, most studies did not record AEs or they did not comprehensively report AE monitoring and the recording procedures followed.^40–42^^,^^65^^,^^66^ Of note, many AEs arose from health issues not associated with exercise. Thus, we suggest a well-defined definition and recording of any AEs in future similar studies to accurately evaluate the safety of interventions that require the long-term commitment of fragile individuals. Regarding this issue, although current guidelines and standard protocols have been developed to help researchers in all biomedical fields to systematically report AEs of experimental interventions,^28^^,^^73^ a specific guideline for reporting AEs associated with physical activity interventions in physical therapy studies would address this important issue.

Moreover, in the studies included in this review, the intensity of the experimental exercise was moderate to high, in accordance with guidelines for exercise in older adults.^74^ Nevertheless, the intensity of the training session in most studies was personalized to the individuals’ capabilities in order to ensure safety and compliance.^40^^,^^41^^,^^43^^,^^48^^,^^64^^,^^65^^,^^66^

### Limitations

This systematic review has some limitations. First, the lack of standardized procedures to measure adherence and AEs may have biased the feasibility and safety estimates of exercise in this population.

With respect to adherence, all the studies included provided a mean cumulative rate, regardless of the type of exercise proposed. Thus, the estimate of patients’ adherence to the prescribed exercise program should be interpreted with caution, given the lack of information concerning the components of exercise being experimented. With respect to AEs, several studies did not report the monitoring procedures for AEs adopted, nor the type or number of AEs that occurred. Thus, an overestimate of safety cannot be ruled out.

Moreover, data regarding time from diagnosis, cancer stage, ADT treatment duration, and concurrent cancer treatments of individuals who participated in the original studies were not thoroughly reported, thus hindering the generalizability of the results of this review.

### Conclusion

Multicomponent exercise implemented according to guidelines for exercise in older men with PCa undergoing ADT^75^ seems feasible. Future research should undertake well-designed clinical trials to assess the effectiveness of high-intensity exercise programs that include structured neuromotor exercises, such as balance, agility, coordination, and cognitive exercises.^74^^,^^75^ Researchers should include standardized methods to record AEs, especially when high-impact exercises (eg, football training) are applied. Moreover, outcome measures should go beyond the measurements of BMD, focusing on the impact of exercise on clinically relevant end points such as the risk of accidental falls and fractures.

## Supplementary Material

PTJ-2020-0872_R2_Supplementary_eAppendix_pzab288

## Data Availability

The datasets generated during and/or analyzed during the current study are available from the corresponding author on reasonable request.

## Systematic Review Registration

The study protocol was registered with the International Prospective Register of Systematic Reviews (PROSPERO) (CRD42020163416).
