# Supplementary material for: Feasibility and Safety of Physical Exercise to Preserve Bone Health in Men With Prostate Cancer Receiving Androgen Deprivation Therapy: A Systematic Review
Source: Phys Ther. 2021 Dec 23;102(3):pzab288. doi: 10.1093/ptj/pzab288 (PMC8970430; doi:10.1093/ptj/pzab288)
Supplement: PTJ-2020-0872_R2_Supplementary_eAppendix_pzab288 [file ptj-2020-0872_r2_supplementary_eappendix_pzab288.pdf]

eAppendix 1- Search strategies

Ovid Medline on 06/07/2021

#1 "Androgen Antagonists"[Mesh] OR "Gonadotropin-Releasing Hormone"[Mesh]

#2 hormone* OR androgen OR androgen deprivation therapy

#3 #1 OR #2

#4 "Prostatic Neoplasms"[Mesh]

#5 prostat* AND (cancer OR tumor OR neoplasm)

#6 #4 OR #5

#7 "Exercise"[Mesh]

#8 "Physical Therapy Modalities"[Mesh]

#9 exercise* OR physical activit* OR physical therap*

#10 #7 OR #8 OR #9

#11 #3 AND #6 AND #10

Embase on 06/07/2021

#1. hormone*:ab,ti OR androgen:ab,ti OR 'androgen deprivation therapy':ab,ti

#2. 'antiandrogen'/exp/mj OR 'gonadorelin derivative'/exp/mj

#3. #1 OR #2

#4. prostat*:ab,ti AND (cancer:ab,ti OR tumor:ab,ti OR neoplasm:ab,ti)

#5. 'prostate tumor'/exp/mj

#6. #4 OR #5

#7. exercise*:ab,ti OR 'physical activit*':ab,ti OR 'physical therap*':ab,ti

#8. 'exercise'/exp/mj OR 'physiotherapy'/exp/mj

#9. #7 OR #8

#10. #3 AND #6 AND #9

CINAHL on 06/07/2021

S1 hormone* OR androgen OR androgen deprivation therapy

S2 (MH "Androgen Antagonists")

S3 (MH "Gonadorelin")

S4 prostat* AND (cancer OR tumor OR neoplasm)

S5 (MH "Prostatic Neoplasms")

S6 (MH "Exercise")

S7 (MH "Physical Therapy")

S8 exercise* OR physical activit* OR physical therap*

S9 S1 OR S2 OR S3

S10 S4 OR S5

S11 S6 OR S7 OR S8

S12 S9 AND S10 AND S11

Cochrane Library on 06/07/2021

#1 MeSH descriptor: [Androgen Antagonists] explode all trees

#2 MeSH descriptor: [Gonadotropin-Releasing Hormone] explode all trees

#3 (hormone* OR androgen OR androgen deprivation therapy):ti,ab,kw

#4 #1 or #2 or #3

#5 MeSH descriptor: [Prostatic Neoplasms] explode all trees

#6 (prostat* AND (cancer OR tumor OR neoplasm)):ti,ab,kw

#7 #5 or #6

#8 MeSH descriptor: [Exercise] explode all trees

#9 MeSH descriptor: [Physical Therapy Modalities] explode all trees

#10 (exercise* OR physical activit* OR physical therap*):ti,ab,kw

#11 #8 or #9 or #10

#12 #4 and #7 and #11
